# The role of integrated psychological support in breast cancer patients: a randomized monocentric prospective study evaluating the Fil-Rouge Integrated Psycho-Oncological Support (FRIPOS) program

**DOI:** 10.1007/s00520-023-07732-4

**Published:** 2023-04-14

**Authors:** Cristina Civilotti, Diana Lucchini, Gianluca Fogazzi, Fabrizio Palmieri, Alice Benenati, Alberto Buffoli, Veronica Girardi, Nella Ruzzenenti, Alessia Di Betta, Edoardo Donarelli, Fabio Veglia, Giulia Di Fini, Gabriella Gandino

**Affiliations:** 1 Department of Psychology, University of Turin, Turin, Italy; 2 Salesian University Institute (IUSTO), Turin, Italy; 3 Breast Psycho-Oncology, EUSOMA-Certified Breast Unit, Istituto Clinico Sant’Anna, Via del Franzone 31, 25127 Brescia, Italy; 4 Associazione Priamo, Via della Lama, 61, 25133 Brescia, Italy; 5 Breast Medical Oncology, EUSOMA-Certified Breast Unit, Istituto Clinico Sant’Anna, Via del Franzone 31, 25127 Brescia, Italy; 6 Breast Surgery, EUSOMA-Certified Breast Unit, Istituto Clinico Sant’Anna, Via del Franzone 31, 25127 Brescia, Italy; 7 Radiation Oncology, EUSOMA-Certified Breast Unit, Istituto Clinico Sant’Anna, Via del Franzone 31, 25127 Brescia, Italy; 8 Breast Radiology, EUSOMA-Certified Breast Unit, Istituto Clinico Sant’Anna, Via del Franzone 31, 25127 Brescia, Italy; 9 Breast Pathology, EUSOMA-Certified Breast Unit, Istituto Clinico Sant’Anna, Via del Franzone 31, 25127 Brescia, Italy

**Keywords:** Breast cancer, Psycho-oncology, Support, Anxiety, Depression, Integrative care

## Abstract

**Purpose:**

This study examined the effects of Fil-Rouge Integrated Psycho-Oncological Support (FRIPOS) in a group of women with breast cancer compared with a group receiving treatment as usual (TAU).

**Methods:**

The research design was a randomized, monocentric, prospective study with three time points of data collection: after the preoperative phase (T0), in the initial phase of treatments (T1), and 3 months after the start of treatments (T2). The FRIPOS group (*N* = 103) and the TAU group (*N* = 79) completed a sociodemographic questionnaire, the Symptom Checklist-90-R (SCL-90-R) at T0; the European Organization for Research and Treatment of Cancer (EORTC) Quality of Life Questionnaire (QLQ) C30 and EORTC QLQ-BR23 at T1; and SCL-90-R, EORTC QLQ-C30, and EORTC QLQ-BR23 at T2.

**Results:**

A series of independent and paired *t* tests showed that patients in the FRIPOS group performed better on all scales related to symptomatic manifestations and on some quality of life scales (fatigue, dyspnea, and sleep disturbances) at T2. In addition, a series of ten multiple regressions were performed to predict each SCL subscale at T2 from the SCL score at T0 and the EORTC QLQ-C30 scores at T2. In nine of ten regression models (all except somatization), both FRIPOS group membership and QoL subscale contributed significantly to prediction.

**Conclusions:**

This study suggests that patients in the FRIPOS group have more benefits in emotional, psychological, and collateral symptoms than patients in the TAU group and that these improvements are due to integrated psycho-oncology care.

## Introduction

Breast cancer is the most commonly diagnosed cancer in Italian women [1]. In 2021, 834,200 women in Italy were living with a breast cancer diagnosis. Although the mortality rate has decreased thanks to early diagnosis and medical advances in the care of women with this disease, and the 5-year survival rate is 87% [2], it is necessary to deepen the topic of psychological adaptation to the disease, which inevitably brings changes in the different areas of a woman’s life [3].

### The psycho-oncological support

Psycho-oncology is a branch of the oncology disciplines that is particularly concerned with two psychological dimensions: the psychological responses of patients and their families to all phases of disease and staff stress and the psychological, social, and behavioral factors that influence cancer onset and disease survival [4]. Psychological support for cancer patients is now recognized as a fundamental aspect of treatment pathways [5], but interventions are often highly targeted and delivered separately from the medical context; examples include cognitive behavioral therapy [6–8], mindfulness and relaxation techniques [9], psychoeducation [10], and family and couples therapy [11]. However, there are few operational models describing how psycho-oncological support interacts with medical staff in acute care, especially in the Italian context [12], and furthermore, there are still many barriers that prevent cancer patients from seeking support.

### Barriers to seeking psychological support in cancer patients

The communication gap between the patient and the medical staff is one of the main problems [13]. In addition, according to the literature, cancer patients often have several unmet needs, with psychological and information problems being the most common [14, 15]. Finally, many patients are not fully aware of their emotional, cognitive, and psychological vulnerability. This may be a temporary condition due to the traumatic effects of the disease, which can lead to a pathological state of numbness [16], or in other cases due to their previous cognitive or emotional functioning [17, 18]. Other common problems are that patients are not adequately informed about support services [19], that they may be perceived as stigmatizing [20], and that medical staff may not be adequately informed about the role of psychosocial care [21].

### Psycho-oncology as an integrated support in routine multidisciplinary cancer care

Whereas in the past, support was usually provided only at the patient’s request, today, the modality has evolved to the so-called tiered models (or stepped models) based on monitoring of suffering [22, 23]. For example, in the UK, the National for Health and Care Excellence (NICE) [24] has developed a psychological intervention pathway with a four-stage paradigm that targets psychological problems or needs through screening and provides treatment as needed.

This approach remains the most widely used and proven, including cost-effectiveness [25], but it has several limitations. First, because the causes of suffering are complex, focusing exclusively on psychological problems risks overlooking the patient’s other perceived needs [14]. In addition, research has shown that most patients who have a high distress screening score with a specific assessment tool do not want to be referred to psychological counseling, whereas patients with a low distress score often hope for some form of support [26, 27]. Finally, some research suggests that an accurate diagnosis of distress is not necessarily linearly related to the ability of health care professionals to control and effectively treat the symptoms that are the cause of distress [28].

An organized method of care that ensures that all of the patient’s needs are met in a coherent and seamless manner is referred to as an “integrated system of care.” Proposals have emerged around the world in the last decade, and although development is ongoing, studies appear promising. These procedures are integrated into clinical routines and address all patients, at least in the initial phase [29, 30].

## The FRIPOS project

The Fil-Rouge Integrated Psycho-Oncological Support (FRIPOS) project is in line with the agreement of April 17, 2019, between the Italian State and its Regions, and the European Cancer Plan presented in February 2021 [31], even if it was designed following the Italian National Cancer Plan of 2016. In it, an integrated supportive intervention based on a close synergy between psycho-oncologists, medical, and nursing staff is proposed [32, 33].

The aim of this study was to evaluate the impact of the FRIPOS model compared with routine care in a sample of women with breast cancer.

### Clinical steps and research procedure

As shown in Table 1, which illustrates the clinical and research steps during the project, the psycho-oncologist was present in the FRIPOS group at various times during cancer treatment.

**Table 1:** Phases of the clinical project and moments of administration of the research project

Clinical phases	Phases of the Fil-Rouge project		Research steps
	TAU group	FRIPOS group	
Preliminary phase	-	-	Administration of informed consent Randomization process
I Diagnostic phase	Discussion with the medical staff (radiotherapist/breast specialist)	Integrated session with medical staff (breast radiologist/psycho-oncologist) Individual discussion with the psycho-oncologist for the management of experiences related to the diagnosis	
II Pre-surgery phase	Discussion with medical staff (radiotherapist/breast specialist/surgeon)	Integrated session with medical staff (breast radiologist/surgeon/psycho-oncologist) Individual discussion with the psycho-oncologist for the emotional management of the pre-surgery experience	T0 Administration of the socio-demographic questionnaire Administration of SCL-90-R
III Initial phase of treatment	Discussion with medical staff (oncologist/radiotherapist)	Integrated session with medical staff (surgeon/radiotherapist/psycho-oncologist) Individual discussion with the psycho-oncologist for the emotional management of the experience related to the treatment phase	T1 Administration of EORTC QLQ-C30 Administration of EORTC QLQ-BR23
IV Follow-up during the treatment	Discussion with medical staff (oncologist/radiotherapist)	Integrated session with medical staff (surgeon/radiotherapist/psycho-oncologist) Individual discussion with the psycho-oncologist for the emotional management of the experience related to the treatment phase	T2 Administration of SCL-90-R Administration of EORTC QLQ-C30 Administration of EORTC QLQ-BR23

The overall clinical goals of the intervention were identified by consulting the clinical scientific literature and can be summarized as follows: (A) encouraging the patient to adapt to the new condition by helping her cope with the physical, psychological, social, and relational changes caused by the disease [34]; (B) deepening the problem areas by identifying the patient’s needs in order to provide integrated and personalized therapeutic interventions [35, 36]; (C) facilitating emotional expression by encouraging the patient to recognize and control anxious and/or depressive states [37]; (D) paying attention to body image to facilitate the process of accepting therapies [38, 39]; (E) communicating the patient’s needs to the treatment team to jointly develop a rehabilitation project for each individual patient [40].

We hypothesized that women who received integrated support would have lower scores for psychopathological symptoms, better psychological and emotional functioning, and better quality of life parameters than the group that received treatment as usual (TAU).

### Ethics and research design

The intervention and the filling in of the questionnaires took place during the hospitalization in the surgical department and during the visit to the oncological day hospital and/or to the “Alte Energie” Center of the Oncological Radiotherapy Department of the Clinical Institute of S. Anna in Brescia — San Donato Group. The project was approved by the Ethics Committee of the Clinical Institute of S. Anna in Brescia (Prot. Number: 2016.1.1; June 6, 2016).

The questionnaires were labeled with an alphanumeric code, the combination of which was known only to the nursing staff responsible for randomization. The medical staff and the researchers who performed the data analysis were blinded to this information.

The research design was a randomized, prospective study with triple data collection according to the steps of the clinical intervention: preoperative phase (T0), initial phase of treatments (T1), and 3 months after the start of treatments (T2) (Table 1).

In the pre-study phase, nurses informed patients of the opportunity to participate in the research project, obtained informed consent, and performed randomization using the online software Research Randomizer (version 4.0). The Symptom Checklist-90-R (SCL-90-R) was used only at T0 and T2 because it was considered a specific and primary tool for evaluating the FRIPOS program by assessing symptom indices at baseline and at the end of the project. At T1 and T2, patients completed two quality of life (QLQ) questionnaires developed by the European Organization for Research and Treatment of Cancer (EORTC), namely C-30 and BR-23.

### Measures

The instruments used are all standardized and validated in the Italian context.

#### Symptom Checklist-90-R

The SCL-90-R [41, 42] is a self-administered questionnaire for the assessment of psychological stress and psychopathological symptoms. It consists of 90 items measuring both a Global Severity Index, a global indicator of the intensity of psychological distress complained of by the respondent, and nine primary symptom dimensions: somatization (SOM), obsessions (O-C), interpersonal sensitivity (I-S), depression (DEP), anxiety (ANX), hostility (HOS), phobic anxiety (PHOB), paranoid ideation (PAR), and psychoticism (PSY).

Subjects’ responses ranged from 0 (not at all) to 4 (very strongly). Cronbach’s *α* was measured on item scores at the two induction time points, with *α* at T0 = 0.96 and *α* at T2 = 0.98.

#### EORTC QLQ-C30 and EORTC QLQ-BR23

The EORTC QLQ-C30 [43] is an instrument for measuring quality of life in cancer patients. In addition to the general quality of life (QoL) assessment, this questionnaire includes the following: five function scales — physical (PF), role (RF), cognitive (CF), emotional (EF), and social (SF) functions; three symptom-related scales — fatigue (FA), nausea and vomiting (NV), and pain (PA); and six individual items on loss of appetite (AP), dyspnea (DY), sleep disturbances (SD), constipation (CO), diarrhea (DI), and financial difficulties (FD). The test includes 30 items with a response range of 1 (none) to 4 (very severe). The QLQ-BR23 is a questionnaire designed to assess the specific problems of breast cancer patients. This particular module includes 23 questions assessing body image (BRBI), sexual functioning (BRSEF), sexual experience (BRSEE), future prospects (BRFU), side effects of systemic therapy (BRST), breast symptoms (BRBS), arm symptoms (BRAS), and hair loss (BRHL). Responses ranged from 1 (none) to 4 (very severe). Cronbach’s *α* of the QLQ-C30 at T1 = 0.80 and T2 = 0.85, whereas the *α* of the QLQ-BR23 at T1 = 0.71 and T2 = 0.78.

### Statistical analyses

Statistical analyses were performed using Statistical Package for the Social Sciences software (SPSS, version 26). A total of 124 subjects (69.67%) answered the entire questionnaire without omissions. Subscales with two or more missing values were not included in the calculation. This procedure resulted in 177 complete questionnaires (98.2%).

To compare the two groups (FRIPOS group and TAU group) analyzed at the same time of administration, multiple independent-samples *t* tests were performed to determine whether there were differences in the subscales of the three instruments at T0 (for SCL-90-R) or T1 (for QLQ-C30 and QLQ-BR23) compared with T2. Then, a series of paired-samples *t* tests were performed to determine whether there was a statistically significant difference between T0 (for SCL-90-R) or T1 (for QLQ-C30 and QLQ-BR23) and T2, in the TAU and FRIPOS groups separately. Finally, a series of 10 multiple regressions were performed after controlling for sociodemographic variables (age, romantic relationship, and presence of sons or daughters) to predict each SCL-90-R subscale at T2 from the SCL-90-R score at T0 and the quality-of-life measure at T2, using the QLQ-C30 scores at T2. We used multiple regression analyses to determine the relative contribution of each predictor (membership in the FRIPOS group vs membership in the TAU group and QoL index) to the total variance explained.

### Sample

Women who had been diagnosed with operable breast cancer were included in the study (ICD-10-CM Diagnosis Code C50.919) [110]. The exclusion criteria for the study were (a) cognitive impairment and/or psychiatric comorbidity and/or a physical condition related to the disease that, in the opinion of the treating physicians or the administrator, could lead to invalid data when completing the questionnaires; (b) poor knowledge of the Italian language; and (c) an assumed life expectancy of less than 6 months at the time of initial diagnosis.

A total of 270 women were recruited for this study, of whom 182 gave informed consent to participate and provided data 3 times (participation rate 60%): 103 belong to the FRIPOS group and 79 to the control group (TAU). The mean age of the sample is 57.88 years (SD = 11.55) and ranges from 25 to 87 years. Table 2 shows the sociodemographic variables of the women who participated in the study.

**Table 2:** Sociodemographic characteristics of the participants

Variables	FRIPOS		TAU		Total	
	*N*	%	*N*	%	*N*	%
Education	*N* = 100		*N* = 78		*N* = 178	
None	1	1.0	1	1.3	2	1.1
Elementary school	19	19.0	12	15.4	31	17.4
Middle school	40	40.0	33	42.3	73	41.0
High school	29	29.0	22	282	51	28.7
University	11	11.0	10	12.8	21	11.8
Employment	*N* = 98		*N* = 78		*N* = 176	
Unemployed	6	6.0	3	3.8	9	5.1
Self-employed	3	3.0	7	9.0	10	5.6
Office worker	20	20.0	15	19.2	35	19.7
Manager	0	0.0	1	1.3	1	0.6
Teacher/researcher	10	10.0	5	6.4	15	8.4
Worker	7	7.0	8	10.3	15	8.4
Housewife	29	29.0	22	28.2	51	28.7
Other	23	23.0	17	21.8	40	22.5
Stable relationship	*N* = 100		*N* = 78		*N* = 178	
No	27	27.0	12	15.4	39	21.9
Yes	73	73.0	66	84.6	139	78.1
Son(s)/daughter(s)	*N* = 100		*N* = 78		*N* = 178	
No	23	23.0	14	17.9	37	20
Yes	77	77.0	64	82.1	141	79.2

## Results

### SCL-90-R

Analysis of the results at T0 in terms of psychopathological symptoms did not reveal a significant difference between the two groups (FRIPOS and TAU) in any of the SCL-90-R scales. However, at T2, there was a statistically significant difference between the two groups in all scales of the SCL-90-R. Specifically, in the FRIPOS group, there were improvements in the following: SOM (0.06, 95% CI, 0.01 to 0.12); O-C (0.20, 95% CI, 0.11 to 0.28); DEP (0.18, 95% CI, 0.04 to 0.17); ANX (0.33, 95% CI, 0.24 to 0.43); HOS (0.10, 95% CI, 0.05 to 0.16); PHOB (0.36, 95% CI, 0.01 TO 0.07); PAR (0.10, 95% CI, 0.04 to 0.16); PSY (0.19, 95% CI, 0.13 to 0.25); and on the GSI of 0.16 (95% CI, 0.11 to 0.21). Using the repeated-measures *t* test, a mean significant worsening on the I-S scale of 0.21 (95% CI, − 0.32 to − 0.09) was found when comparing T0 and T2 in the TAU group (Table 3).

**Table 3:** Results at T0 and T2 and comparisons (between FRIPOS and TAU groups at T2 and for repeated measures in each group) on mean SCL-90-R scores

	*T0*				*T2*				*T0-T2 comparison*		
	TAU		FRIPOS		TAU		FRIPOS			TAU	FRIPOS
	M	DS	M	DS	M	DS	M	DS	*t* (between groups at T2)	*t* (repeated measures)	*t* (repeated measures)
SOM	.58	.55	.50	.47	.68	.47	.44	.49	3.372**	− 1.319	2.007*
O-C	.48	.55	.54	.60	.50	.48	.34	.52	2.149*	− .348	4.606**
I-S	.18	.25	.27	.38	.39	.50	.16	.28	3.879**	− 3.582**	3.62**
DEP	.60	.56	.59	.58	.66	.52	.41	.48	3.327**	− 1.008	3.818**
ANX	.59	.54	.64	.60	.59	.50	.31	.39	− 4.338**	.021	7.143**
HOS	.24	.28	.25	.27	.33	.43	.15	.28	− 3.459**	− 1.73	3.576**
PHOB	.14	.26	.97	.18	.19	.31	.61	.13	− 3.945**	− 1.319	2.057*
PAR	.22	.31	.23	.39	.27	.39	.13	.27	2.897*	− 1.16	3.529**
PSY	.28	.33	.34	.35	.31	.35	.15	.25	3.424**	− .579	6.429**
GSI	.42	.35	.44	.38	.49	.40	.28	.32	3.988**	− 1.502	6.226**

Symptoms Checklist subscale scores are reported as mean (M) and standard deviation (SD). For each subscale, the score varies from a minimum of zero to a maximum of four

*SOM* somatization, *OC* obsessive–compulsive, *I-S* interpersonal sensitivity, *DEP* depression, *ANX* anxiety, *HOS* hostility, *PHOB* phobic anxiety, *PAR* paranoid ideation, *PSY* psychoticism, *GSI* global index severity, *T t*-test

^*^p < .05; **p < .01

### EORTC QLQ-C30

Analysis of the data collected with the QLQ-C30, shown in Table 4, indicates that there were no statistically significant differences between the FRIPOS group and the TAU group at T1, making the two subgroups comparable.

**Table 4:** Results at T1 and T2 and comparisons (between FRIPOS and TAU groups at T2 and for repeated measures in each group) on mean EORTC QLQ-C30 scores

	*T1*					*T2*				*T1-T2 comparison*		
	TAU		FRIPOS		TAU			FRIPOS			TAU	FRIPOS
	M	DS	M	DS		M	DS	M	DS	*t* (between groups at T2)	*t* (repeated measures)	*t* (repeated measures)
QL	64.02	19.27	64.68	2.14		58.96	18.33	68.64	19.33	3.410**	2.75**	− 1.846
Functioning subscales												
PF	87.08	14.57	87.32	13.21		82.95	14.85	85.61	15.57	1.159	3.293**	1.114
RF	84.40	2.69	83.99	19.98		82.69	17.70	82.83	22.41	.047	.775	.438
EF	72.43	2.14	75.66	19.49		68.98	18.58	81.18	15.70	4.771**	1.821*	− 2.676**
CF	85.02	17.21	87.62	17.26		84.81	17.33	87.95	18.27	1.171	.103	− .172
SF	85.65	17.03	89.43	16.78		82.27	19.12	9.75	17.71	3.078*	1.616	− .627
Symptom subscales												
FA	28.27	19.79	29.15	19.55		34.73	18.17	27.06	21.85	− 2.515*	− 3.046**	1.055
NV	5.27	12.94	5.44	12.04		8.64	15.52	5.94	12.60	− 1.292	− 1.839	− .332
PA	17.72	23.01	15.67	16.46		18.98	19.00	17.82	22.26	− .371	− .505	− .854
DY	11.39	19.89	9.90	17.34		18.56	18.29	9.57	2.72	− 3.04*	− 2.693	.148
SL	29.11	24.67	25.74	29.39		33.33	25.03	24.42	27.84	− 2.226*	− 1.486	.498
AP	8.01	14.33	9.57	19.62		1.12	17.17	6.27	16.80	− 1.513	− 1.043	1.451
CO	15.61	23.16	16.50	27.73		15.18	21.21	15.84	24.76	.186	.179	.261
DI	3.37	11.44	3.96	11.82		6.32	14.19	3.63	11.45	− 1.412	− 1.622**	.241
FD	4.26	13.05	8.87	19.02		5.94	18.50	1.98	23.10	− 1.623	− .928	− .869

EORTC QLQ-C30 scales and subscale scores are reported as mean (M) and standard deviation (SD)

*QL* global health status/QoL, *PF* physical functioning, *RF* role functioning, *CF* cognitive functioning, *EF* emotional functioning, *SF* social functioning, *FA* fatigue, *NV* nausea and vomiting, *PA* pain, *AP* loss of appetite, *DY* dyspnea, *SL* sleep problems, *CO* constipation, *DI* diarrhea, *FD* financial difficulties, *t t*-test

^*^p < .05; **p < .01

An independent comparison of the two groups at T2 shows that the FRIPOS group performed better on both the QoL scale and the EF scale. Longitudinal comparison (by paired *t* test analyses) between the groups shows that participation in the FRIPOS project resulted in an improvement on the EF scale of 5.53 (95% CI, − 9.62 to − 1.43), whereas in the TAU group, there was a worsening of 3.45 (95% CI, 0.32 to 7.21), a worsening on the QoL scale of 5.06 (95% CI, 1.40 to 8.73), and on the PF subscale of 0.4.14 (95% CI, 0.16 to 6.63).

In addition, there was evidence of treatment benefit in some of the subscales related to physical symptoms: Patients in the FRIPOS group showed better scores on the indices FA, DY, and SL. There was also a significant worsening of 0.295 (95% CI, − 6.58 to − 0.67) in the DI subscale.

### EORTC QLQ-BR23

For the QLQ-BR23 scales specifically related to breast cancer listed in Table 5, patients in the TAU group worsened on both the BRBI scale (8.12 (95% CI, 3.69 to 12.55)) and the BRFU scale (8.02 (95% CI, 2.51 to 13.22)), which was not the case for patients who participated in the FRIPOS project group. Here, there was no significant worsening of the scores and even an improvement in the BRFU score of 9.57 (95% CI, − 15.55 to − 3.58).

**Table 5:** Results at T1 and T2 and comparisons (between FRIPOS and TAU groups at T2 and for repeated measures in each group) on mean EORTC QLQ-BR23 score

	*T1*				*T2*				*T1-T2 comparison*		
	TAU		FRIPOS		TAU		FRIPOS			TAU	FRIPOS
	M	DS	M	DS	M	DS	M	DS	*t* (between groups at T2)	*t* (repeated measures)	*t* (repeated measures)
BRBI	83.54	2.88	85.39	2.15	75.42	24.34	85.64	2.00	3.092*	3.648**	− .138
BRSEF	13.29	2.03	15.83	2.29	14.76	2.14	15.16	2.66	.081	− .98	.383
BRSEE	45.33	23.33	49.38	21.42	46.66	23.57	45.67	2.97	.119	− .253	1
BRFU	64.97	27.16	58.41	31.41	56.96	27.81	67.98	25.35	2.774*	2.898**	− 3.174**
Symptom subscales											
BRST	18.31	13.48	14.05	13.78	26.32	17.11	15.04	16.26	− 4.513**	− 4.052**	− .562
BRBS	16.27	18.18	13.86	14.00	21.37	2.43	15.92	17.48	− 1.928	− 2.889**	− .96
BRAS	15.61	18.10	13.20	16.60	21.23	21.06	16.61	18.63	− 1.561	− 2.546*	− 1.551
BRHL	28.20	22.95	41.66	31.91	33.33	33.33	41.66	41.94	− .685	− .693	0

The results on EORTC QLQ-BR23 are given as mean values of the values recalibrated on a base scale of 100 and standard deviations. EORTC QLQ-BR23 scales and subscale scores are reported as mean (M) and standard deviation (SD)

*BRBI* body image, *BRSEF* sexual functioning, *BRSEE* sexual experience, *BRFU* future prospects, *BRST* systemic therapy side effects, *BRBS* breast symptoms, *BRAS* arm symptoms, *BRHL* upheaval for hair loss, *t t*-test between groups

^*^p < .05; **p < .01

For the symptom scales, inclusion of patients in the FRIPOS group resulted in significant stability in all subscales, whereas there was significant deterioration only in the TAU group, in the BRST scale of 8.02 (95% CI, − 11.95 to − 4.07), the BRBS scale of 5.10 (95% CI, − 8.61 to − 1.58), and the BRAS scale of 5.63 (95% CI, − 10.02 to − 1.23).

### The role of integrated support and quality of life in explaining symptomatology at T2

A series of multiple regressions were performed to predict each subscale of the SCL-90-R at T2 from each subscale of the SCL-90-R at T0 and the QLQ-C30-derived QL scores at T2. In all regression analyses, linearity was assessed by partial regression plots and a plot of student residuals against predicted scores. Independence of the residuals was demonstrated by a Durbin-Watson statistic with values of 1.476 (hostility subscale) and 2.134 (somatization subscale). Homoscedasticity was determined by visual inspection of a plot of student residuals compared with unstandardized predicted values. There was no evidence of multicollinearity, as judged by tolerance values greater than 0.1. The multiple regression model for somatization statistically significantly predicted somatization at *F*(6, 170) = 23.545, *p* < 0.001, adj. *R*^2^ = 0.44. The same was true for the following subscales: O-C, with *F*(6, 170) = 24.000, *p* < 0.001, adj. *R*^2^ = 0.44; I-S, with *F*(6, 170) = 9.044, *p* < 0.001, adj. *R*^2^ = 0.22; DEP, with *F*(6, 170) = 23.496, *p* < 0.001, adj. *R*^2^ = 0.43; ANX, with *F*(6, 170) = 22.541, *p* < 0.001, adj. *R*^2^ = 0.42; HOS, with F(6, 170) = 8.819, *p* < 0.001, adj. *R*^2^ = 0.21; PHOB, with *F*(6, 170) = 9.672, *p* < 0.001, adj. *R*^2^ = 0.23; PAR, with *F*(6, 170) = 13.517, *p* < 0.001, adj. *R*^2^ = 0.30; PSY, with *F*(6, 170) = 17.806, *p* < 0.001, adj. *R*^2^ = 0.36. In addition, the multiple regression model for the Global Severity Index statistically significantly predicted the value of this index at T2, *F*(6, 170) = 24.503, *p* < 0.001, adj. *R*^2^ = 0.45. In nine of ten regression models, both FRIPOS group membership and the QoL subscale significantly contributed to prediction, with a significance level of at least *p* < 0.05. Somatization symptomatology was the only subscale for which FRIPOS group membership did not make a statistically significant contribution to prediction, whereas the QoL subscale did. Regression coefficients and standard errors are provided in Table 6.

**Table 6:** Multiple regression results for each subscale of SCL-90-R scores at T2

*Models*	*B*	95% CI for *B*		SE B	*β*	*R*^2^	Δ *R*^2^
		LL	UL				
Model for SOM						.45	.44
Constant	1.24	.81	1.66	.22			
SOM at T0	.40	.29	.51	.06	.41***		
FRIPOS/TAU	− .07	− .18	.05	.06	.26		
QoL at T2	− .01	− .01	− .01	.00	− .44***		
Model for O-C						.46	.44
Constant	.56	.11	1.02	.23			
O-C at T0	.52	.42	.62	.05	.59***		
FRIPOS/TAU	− .12	− .24	.00	.06	− .12*		
QoL at T2	− .01	− .01	− .01	.00	− .22***		
Model for I-S						.24	.22
Constant	.57	.15	1.00	.22			
I-S at T0	.40	.22	.57	.09	.33***		
FRIPOS/TAU	− .20	− .32	− .09	.06	− .07***		
QoL at T2	− .00	− .01	− .00	.00	− .21**		
Model for DEP						.45	.43
Constant	.85	.37	1.3	.24			
DEP at T0	.45	.34	.56	.06	.50***		
FRIPOS/TAU	− .15	− .27	− .02	.06	− .14*		
QoL at T2	− .01	− .01	− .01	.00	− .31***		
Model for ANX						.44	.42
Constant	.66	.23	1.08	.22			
ANX at T0	.40	.30	.50	.05	.50***		
FRIPOS/TAU	− .22	− .33	− .11	.06	− .24***		
QoL at T2	− .01	− .01	− .00	.00	− .29***		
Model for HOS						.24	.21
Constant	.35	− .03	.73	.19			
HOS at T0	.43	.25	.61	.09	.33***		
FRIPOS/TAU	− .13	− .23	− .03	.05	− .18*		
QoL at T2	− .00	− .01	− .00	.00	− .23**		
Model for PHOB						.25	.23
Constant	.29	.06	.52	.12			
PHOB at T0	.33	.19	.47	.07	.32***		
FRIPOS/TAU	− .08	− .14	− .02	.03	− .17*		
QoL at T2	− .00	− .01	− .00	.00	− .25***		
Model for PAR						.32	.30
Constant	.39	.06	.72	.17			
PAR at T0	.45	.33	.58	.06	.49***		
FRIPOS/TAU	− .11	− .20	− .03	.05	− .17*		
QoL at T2	− .00	− .00	.00	.00	− .12		
Model for PSY						.39	.36
Constant	.37	.07	.67	.15			
PSY at T0	.46	.34	.58	.06	.51***		
FRIPOS/TAU	− .15	− .23	− .07	.04	− .24***		
QoL at T2	− .00	− .01	− .00	.00	− .16*		
Model for GSI						.46	.45
Constant	.58	.24	.93	.17			
GSI at T0	.50	.38	.62	.06	.49***		
FRIPOS/TAU	− .15	− .24	− .06	.04	− .20**		
QoL at T2	− .01	− .01	− .00	.00	− .31***		

*Model* “Enter” method in SPSS Statistics, *B* unstandardized regression coefficient, *CI* confidence interval, *LL* lower limit, *UL* upper limit, *SE B* standard error of the coefficient, *β* standardized coefficient, *R*^*2*^ coefficient of determination, *Δ R*^*2*^ adjusted *R*^2^, *SOM* somatization, *O-C* obsessive–compulsive, *I-S* interpersonal sensitivity, *DEP* depression, *ANX* anxiety, *HOS* hostility, *PHOB* phobic anxiety, *PAR* paranoid ideation, *PSY* psychoticism, *GSI* global index severity

^*^p < .05; **p < .01

## Discussion

The integrated approach proposed in the FRIPOS project seems to be an adequate response to the claims that appear in the literature related to the concept of holistic management [44, 45] and joins the studies that have already demonstrated its effectiveness and importance [46–48].

Indeed, the inclusion of the psycho-oncologist in the treatment has brought significant benefits or at least a state of stability in many areas (in contrast to what happened in the group TAU), especially in relation to several aspects highlighted in the literature: the development of symptomatic manifestations related to psychopathology as a result of or during the journey against cancer and the indices of psychological functioning, especially at the emotional level [49]. Specifically, improvements in symptomatic manifestations (somatization, obsessiveness, depression, anxiety, hostility, phobic anxiety, paranoid ideation, and psychoticism), emotional functioning, and future outlook were observed in the FRIPOS group. The significantly worse score on the I-S scale in the TAU group could be related to the challenges of a cancer diagnosis at the interpersonal level, such as changes in body image and loss of femininity, or at the level of the life-threatening event [50]. The personal support in the FRIPOS group might have helped patients to cope with and regulate feelings of inadequacy, inferiority, and discomfort in interpersonal interactions, but because of the complexity of the physical and psychological correlates of breast cancer, this aspect needs further investigation.

In addition, the results show the effectiveness of the integrated intervention in terms of quality-of-life scales, especially in terms of fatigue, dyspnea, and sleep disturbances. This result can be taken as an indication of the importance of assessing not only the level of distress, but a more comprehensive picture of the person (including the assessment of unmet needs), taking into account in particular nonverbal behaviors as part of a holistic approach that can be of greater use for interventions in breast cancer patients. One of the advantages of the FRIPOS personalized approach is that it can be adapted to the specific needs of the patient. The psycho-oncologist throughout the oncological path can therefore implement psychological support on the basis of the particular psychological functioning of the person. In line with the literature [51, 52], starting from clinical observation, we found that the patients’ needs concerned emotional health, continuity of care, information, adverse effects on their own body and mind, information, and social support. The composition of these needs in each patient varied on the basis of age, stage of treatment, social status, and family composition.

In nine of ten regression models for psychological symptomatology (all except the somatization subscale), membership in the FRIPOS group together with the quality-of-life subscales significantly contributed to the prediction of the mean score of the respective subscale at T2. These results suggest that the FRIPOS program was effective, confirming the hypothesis and joining the ranks of studies demonstrating the importance of integrated approaches [48]. Furthermore, the regression analyses underscore the importance of monitoring quality of life in relation to the potential psychopathological consequences of breast cancer diagnosis and cancer-related treatments, as called for by the scientific community [53]. An integrated approach also allows the psycho-oncologist to continuously and directly assess the patient's psychophysical state, as unmediated observation allows for the capture of all implicit stress signals that cannot always be accurately captured by conventional stress measurement tools [54]. Another strength of this integrated support is that it addresses all patients indiscriminately, i.e., both those who are unable or unwilling to consciously ask for help [55] and those who face unmet needs and find a setting in which to talk about their problems. The integrated intervention does not take the form of a psychological takeover through coercion, but by offering a resource that the patient can access directly according to the perceived needs, an intervention that can adapt to the complexity of each case, and the heterogeneity of the types of patients [56, 57].

Further studies on the cost-effectiveness of an integrated approach are needed, especially for health systems such as the Italian one, which faces an increasing demand for integrated psycho-oncology services but also dwindling financial resources, especially for psychosocial care.

Despite methodological limitations such as the relatively small sample size, the use of self-reports, and a possible implicit influence of the greater participation in the FRIPOS group (in which the women who completed all three surveys were more numerous), this study may provide important general insights for the clinical setting, including various health situations in which the presence of a parallel psychological support network in conjunction with medical interventions could significantly affect the quality of life and psychophysiological balance of those involved in the treatment process. Another limitation is that due to the pioneering and exploratory nature of this project, there is not yet a manualization of the integrative interventions. For this reason, the intervention was planned using information from the scientific literature on actual difficulties reported by cancer patients (especially breast cancer patients), but no measures were taken to ensure consistency of treatment. This problem needs to be addressed in the future. Potential psychological variables that would be useful to examine in future research involve perceived support by family and partner and specific personality traits.

## Conclusions

The FRIPOS project aims to create a holistic pathway for the psycho-oncological care of cancer patients. It also aims to provide Italian health policy makers with a solid decision-making basis for the timely introduction of integrated psycho-oncology services in the Italian health system. In this study, the involvement of a psycho-oncologist during cancer treatment was found to be crucial for breast cancer patients’ psychological well-being and coping with several aspects related to the disease. To improve the situation of women with breast cancer (as well as other cancer patients), it is desirable that psychological support be offered to provide patients with a flexible, integrated, and adaptable treatment pathway for each person’s specific individuality.

## Data Availability

The data presented in this study are available on request from the corresponding author. The data are not publicly available due to privacy reasons.
